# Chronic multiple lung abscesses presenting as a Left lung apical mass: a ticking bomb?

**DOI:** 10.11604/pamj.2014.17.292.3081

**Published:** 2014-04-17

**Authors:** Godfrey Mutashambara Rwegerera, Erasmus Kago Taolo

**Affiliations:** 1 School of Medicine, University of Botswana and Physician at Princess Marina Hospital, Gaborone, Botswana; 2 Department of Internal Medicine, Princess Marina Hospital, Gaborone, Botswana

**Keywords:** Lung abscess, apical mass, lung tumour

## Image in medicine

A 32 years-old female, HIV positive who defaulted using Atripla for over a period of one year with current CD4 of 86/µL was referred to a tertiary hospital with chest x-ray showing a lesion suspicious of a lung tumour. She had presented at local hospital three (3) months earlier with non-productive cough, associated with left sided stabbing chest pains and fevers, and treated as a case of community acquired pneumonia. The cough and fevers subsided. Past medical history is remarkable for Pulmonary Tuberculosis in 2004. The patienthas been afebrile throughout her six weeks hospital stay. General examination revealed no respiratory distress, mild pallor and moderate finger clubbing with temperature of 36.70C. Other vitals were within the normal range. Respiratory rate of 19breaths/minute, decreased air entry in the left lower zone posteriorly, left basal coarse crepitations, left upper zone dullness on percussion with bronchial breath sounds. The rest of systemic examination was not remarkable. Full blood count showed normal WBC = 6.12K/ul and Neutrophil = 4.9K/ul, moderate anemia with Hb = 7.4g/dl, MCV = 70.1fl and Platelet = 283X 109/l. Pus results did not reveal any organisms on culture; Ziehl-Nelson for AFB was negative. Cytology results showed acute and chronic inflammatory cells, with no evidence of malignancy. Computerized tomography of the chest revealed multiple left lung abscesses, Bronchiectasis and fibrosed left middle hemithorax. In view of multiple lung abscesses complicated with bronchiectasis, the cardiothoracic surgeon has planned an elective surgical intervention to drain the abscesses and a pneumonectomy.

**Figure 1:**
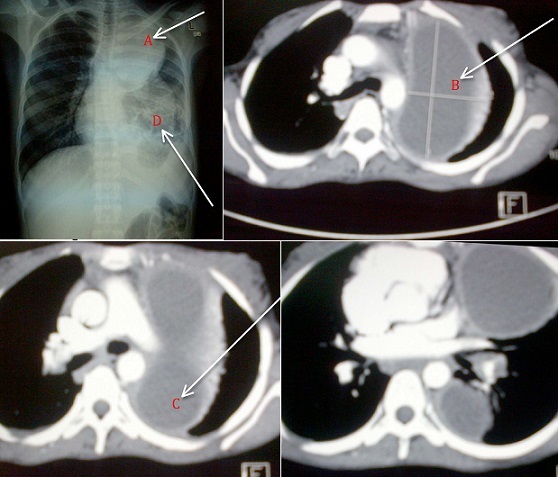
Posterior-anterior chest x-ray view showing a well circumscribed oval opacification resembling a lung mass (A), Contrasted computerized tomography (CT) of the chest showing the largest lung abscess measuring 10.75x5.93cm (B), Contrasted chest CT scan showing a lung abscess at the base of the left lung, Posterior-anterior chest x-ray view showing bronchioectatic changes in a left collapsed/fibrosed left middle lobe (D).

